# My Interventional Drug-Eluting Stent Educational App (MyIDEA): Patient-Centered Design Methodology

**DOI:** 10.2196/mhealth.4021

**Published:** 2015-07-02

**Authors:** Andrew Dallas Boyd, Kaitlin Moores, Vicki Shah, Eugene Sadhu, Adhir Shroff, Vicki Groo, Carolyn Dickens, Jerry Field, Matthew Baumann, Betty Welland, Gerry Gutowski, Jose D Flores Jr, Zhongsheng Zhao, Neil Bahroos, Denise M Hynes, Diana J Wilkie

**Affiliations:** ^1^ Department of Biomedical and Health Information Sciences University of Illinois at Chicago Chicago, IL United States; ^2^ Biomedical Informatics Core Center for Clinical and Translational Sciences University of Illinois at Chicago Chicago, IL United States; ^3^ Division of Cardiology Deparment of Medicine University of Illinois at Chicago Chicago, IL United States; ^4^ Department of Pharmacy Practice University of Illinois at Chicago Chicago, IL United States; ^5^ Department of Biobehavioral Health Science University of Illinois at Chicago Chicago, IL United States; ^6^ University of Illinois at Chicago Chicago, IL United States; ^7^ Office for the Vice Chancellor for Research University of Illinois at Chicago Chicago, IL United States; ^8^ Division of Health Promotion Research Department of Medicine University of Illinois at Chicago Chicago, IL United States; ^9^ VA Information Resource Center and the Center of Innovation for Complex Chronic Healthcare Health Services Research and Development Service Edward Hines, Jr. VA Hospital Maywood, IL United States

**Keywords:** drug-eluting stents, health informatics, Kolb's learning theory, mHealth, patient-centered design, patient education

## Abstract

**Background:**

Patient adherence to medication regimens is critical in most chronic disease treatment plans. This study uses a patient-centered tablet app, “My Interventional Drug-Eluting Stent Educational App (MyIDEA).” This is an educational program designed to improve patient medication adherence.

**Objective:**

Our goal is to describe the design, methodology, limitations, and results of the MyIDEA tablet app. We created a mobile technology-based patient education app to improve dual antiplatelet therapy adherence in patients who underwent a percutaneous coronary intervention and received a drug-eluting stent.

**Methods:**

Patient advisers were involved in the development process of MyIDEA from the initial wireframe to the final launch of the product. The program was restructured and redesigned based on the patient advisers’ suggestions as well as those from multidisciplinary team members. To accommodate those with low health literacy, we modified the language and employed attractive color schemes to improve ease of use. We assumed that the target patient population may have little to no experience with electronic tablets, and therefore, we designed the interface to be as intuitive as possible.

**Results:**

The MyIDEA app has been successfully deployed to a low-health-literate elderly patient population in the hospital setting. A total of 6 patients have interacted with MyIDEA for an average of 17.6 minutes/session.

**Conclusions:**

Including patient advisers in the early phases of a mobile patient education development process is critical. A number of changes in text order, language, and color schemes occurred to improve ease of use. The MyIDEA program has been successfully deployed to a low-health-literate elderly patient population. Leveraging patient advisers throughout the development process helps to ensure implementation success.

## Introduction

### Overview

Patient medication adherence is challenging across all health care domains [1]. One area where nonadherence bears a high mortality risk is after a percutaneous coronary intervention (PCI). PCI is the most common form of revascularization performed worldwide for the treatment of hemodynamically significant coronary artery stenosis, with over 1 million procedures performed in the United States every year [2]. PCI is performed when the arteries have an increased amount of plaque or have become narrowed. In its most common practice, the cardiologist places a drug-eluting stent (DES) at the site of the narrowing to displace the plaque from against the wall of the artery, which results in improved blood flow. For a year after undergoing the procedure, patients must adhere to a strict drug regimen of 2 antiplatelet medications taken once a day, every day for a year. A previous study [3] noted that 1 of 7 myocardial infarction patients who received a DES during a cardiac catheterization stopped taking their dual antiplatelet therapy (DAPT) within 30 days of the procedure. This failure leads to a *9 times greater* risk of death in the following year due to stent thrombosis [3-6]. DAPT discontinuation rates are 14-57% [7-9]. Multiple authors have called for research improving adherence to DAPT [10,11], with recognition that patient education is one of the few modifiable factors to reduce risks after a PCI [3,12].

### Use of Mobile Technology to Educate Patients

Mobile technology provides a unique opportunity to engage and educate PCI patients about the importance of DAPT. However, technology alone cannot improve patient education, especially education related to patient behavior. However, incorporating learning theories into patient-centered educational material is one way to improve this scenario [13-15]. Indeed, Kolb’s experiential learning theory [13] has been successfully applied to patient education in a number of health-related areas [16-24]. Kolb’s theory is notable for its 4-stage learning circle to engage learners who have different learning styles. A Kolb’s learning circle includes (1) a concrete experience, (2) reflective observations, (3) abstract conceptualization, and (4) active experimentation.

In addition to the health care need and the educational theories, another area unique to technology is designing tools to meet the needs of the users. Designing technology interfaces with user participation has been performed for a number of years [25]. The concept of human-centered interfaces has become so standardized that the International Standardization Organization (ISO) has created a standard to ensure the ergonomics of interactive systems [26]. However, the vast majority of patient-centered educational materials are still paper based, with a limited number of studies evaluating electronic patient educational materials [27]. Most patient-centered software app development has focused on the technical correctness of the algorithm [28]. However, collaborative approaches to biomedical communication have long been advocated [29]. The unique field of health-related patient software using biomedical communication techniques has not applied the concepts from the merging of the fields. We found nothing in the literature that explains the methodology as applied to interactive patient education using a participatory design process. Working with a team, all of whom have had experience with postcardiac rehabilitation, the goal was to explain the process in both technology terms and patient-care phrases. The objective of this paper is to describe the design, methodology, limitations, and results about the ability of the patients to use this tablet app as measured by time of completion and interactive recordings of an electronic mobile technology-based patient education app designed to improve DAPT adherence.

## Methods

### Development of the Drug Adherence Electronic Tablet App

A multidisciplinary health care team of cardiologists, pharmacologists, patient educators, nurses, and health informatics professionals assisted in development of a Kolb’s theory-based drug adherence electronic tablet app called “My Interventional Drug-Eluting Stent Educational App (MyIDEA).” This tablet app was meant to be a stand-alone educational tool to supplement the education of the nurse and physician in medication adherence following the procedure. The team completed a literature review on the DES and the PCI procedure. Initially, a biomedical illustrator (KM) met with the team members and asked for their input about concepts and objectives for DAPT for DES patients. In addition, the biomedical illustrator observed that both the nurse and physician gave verbal instructions to the patient population of interest. She integrated the observed concepts into the development of the app. The team also reviewed the printed educational material typically sent home with a DES patient.

Patient viewpoints and engagement were crucial in the app development. To identify patients who were willing and able to share input, the research team reached out to the local Chicago chapters of Mended Hearts. Mended Hearts is a national nonprofit organization that offers the gift of education, information, hope, and encouragement to heart disease patients, as well as to their families and caregivers. The team was looking for active members who would like to assist in the development a patient-centered educational app. A total of 5 patient advisers agreed to participate. Over several meetings, they gave input into the development based on their personal experiences. Based on the input from our team and the patient advisers, 4 crucial attributes of the target patient population were identified, including (1) low health literacy, (2) aged older than 50 years, (3) able to speak English, and (4) received a PCI with a DES at the University of Illinois Hospital. Indeed, the typical DES patient may be affected by low literacy, have low health literacy, and little to no higher education as well as an average age of about 65 years [30].

### Wireframes and Final App Development

The educational content for the app was first created as a primitive wireframe. A wireframe is a simple schematic similar to a blueprint of a house where major concepts can be discussed before formal development is initiated. Over a 4-month period, we created 6 different versions of the app wireframe and enhanced them with collective observations and collaboration. We showed a paper version of the wireframe to 3 patients in an Institutional Review Board (IRB)-approved study in the hospital for additional feedback (2009-0711) using a semistructured interview.

After agreement on the final HTML5 design of the app, we created static HTML pages for the tablet. The static Web pages could not be customized to individual patients but simulated a single interaction for a demonstration patient.

After vetting the static Web pages, an investigation into platforms occurred. Because of some technical limitations of the iOS platform, the software engineer recommended a switch to the Android platform. He also recommended building in multilingual capability and replacing the graphical text with actual text so that it could be changed as needed without requiring recompilation.

The complete system consists of a hybrid HTML5 Android tablet app, a Health Insurance Portability and Accountability Act secure patient-tracking and data portal, a human-computer interaction monitoring “click-tracking” server, an audio upload server, and a configuration control server for dynamically reconfiguring or updating the text and formatting the app. The tablet app has 2 audio components: narration and recording and playback of the patient’s impressions. The text of the program was written at a sixth-grade reading level. The narration provides an additional method for individuals with low literacy to understand the text-based information, which is at a sixth-grade level. Reflective observation was one of the key aspects of learning in the Kolb’s theory [13]. We have the audio recording and playback to gain a better understanding of what the patients are thinking, how they verbalize concerns, and solve problems based on their reflections of overcoming their obstacles. All audio components contained words and phrases that can be readily understood.

After the MyIDEA program was fully functional, the team and all patient advisers reviewed it. A few modifications were then made including the creation of a summary or recap screen before the learner could exit the app and captioning of instructions on the interface tutorial screens for scenarios where the app was muted. An IRB-approved pilot randomized control trial was initiated, with the interventional arm having access to the functional app. Here, we report the results of the design process overview, which was presented in the earlier section, and initial time utilization of MyIDEA for the interventional arm.

## Results

### Framework of the App

**Figure 1:**
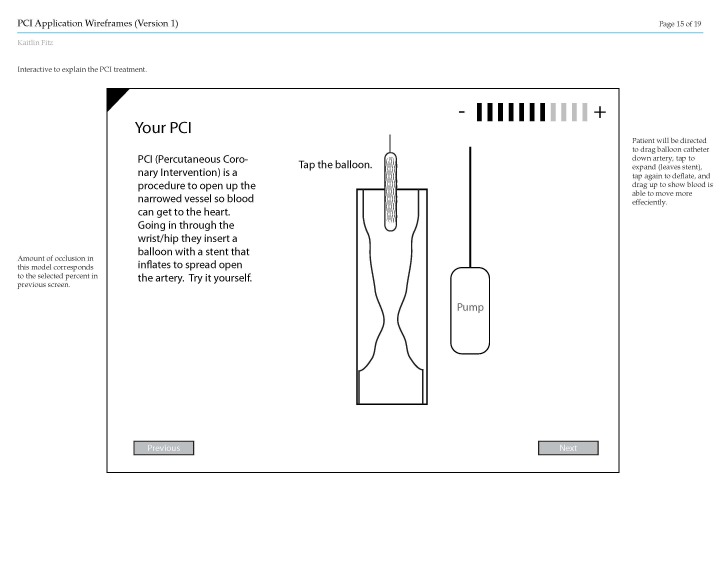
Sample page from the initial wireframe shown to the multidisciplinary team and patient advisers for feedback.

When the patient advisers and development team first met, we discovered that none of the advisers had any experience with software development, which necessitated description of the purpose of the wireframe (Figure 1) and the envisioned educational app. One of the novel aspects of the patient-centered education was customizing the program to the findings of the individual patient, such as symptoms, medication, and procedure findings. The advisers indicated that the pictures and draft diagrams were too vague for meaningful critique. They indicated that the program should also include an audio component, that the app objectives for medication adherence needed to be explained in the initial screen, and that the duration of DAPT needed to be clearly stated.

The framework of the app was built integrating Kolb’s experiential learning theory. This theory is a 4-part cyclical model aiming to address 4 types of learning styles: converger, diverger, assimilator, and accommodator [13]. Kolb’s 4-stage learning cycle shows how experience can be translated into concepts. The 4 stages are concrete experience, reflective observations, abstract conceptualization, and active experimentation [13].

**Figure 2:**
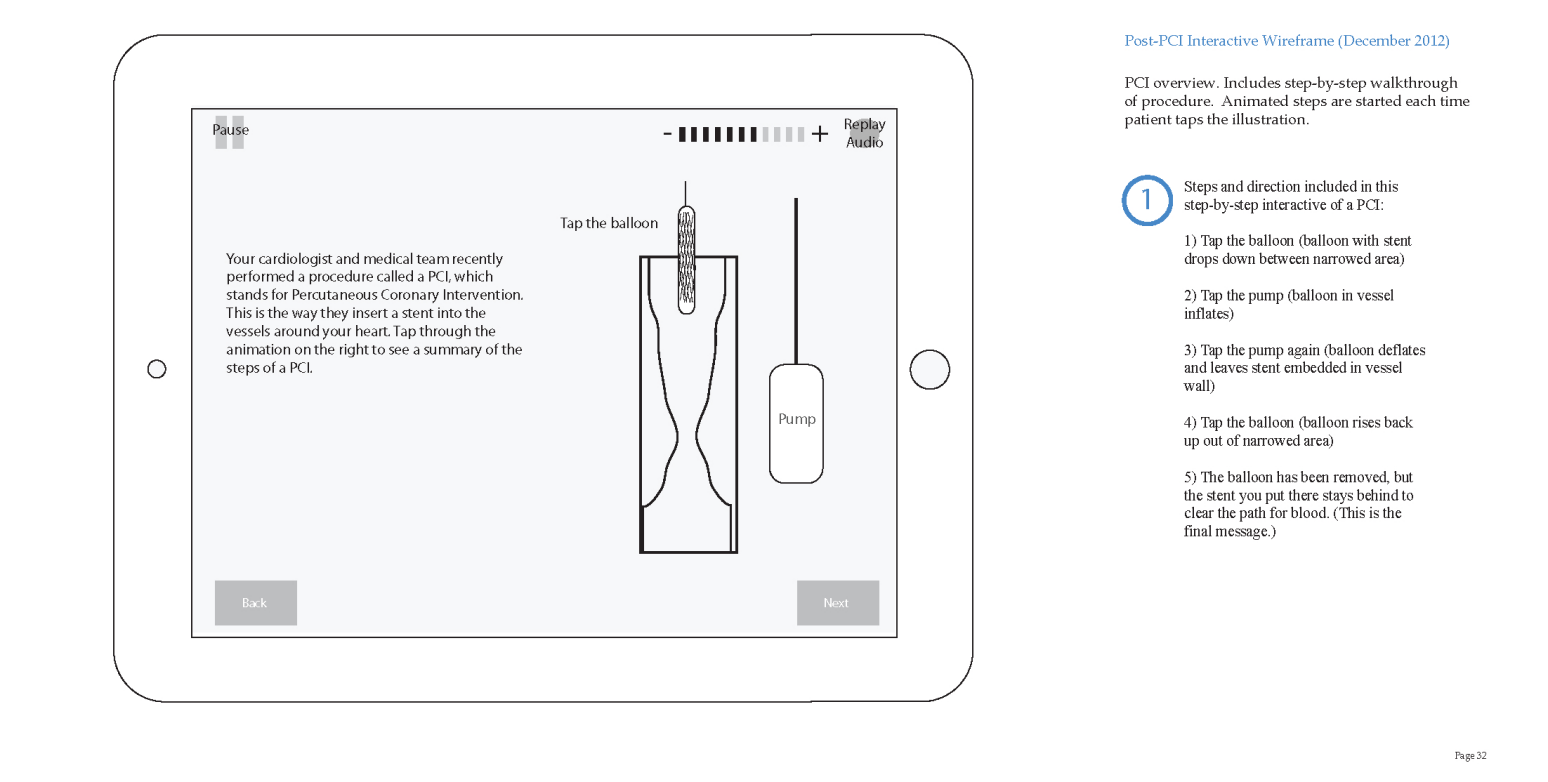
Wireframe number 2 sample page about how a stent works.

A second iteration (Figure 2) of the wireframes was created and discussed with patient advisers and team members during a second meeting. This discussion revealed that the overall message of the purpose of the DAPT was not portrayed adequately. The patient advisers suggested that the main message be integrated throughout the app with persuasive reasons for adherence clearly demonstrated, instead of only giving the information at the conclusion. Another identified issue was that the wireframes did not adhere sufficiently to Kolb’s theory. In response, we added patient stories and reflective observations about the stories, along with the patient’s symptoms to ensure integration of the entire Kolb’s learning circle, which was a large-scale restructuring of the app. This procedure offered reinforcement of several critical points, information on proper postcardiac medication regimen, rapid communication with the physician’s staff, and a comfort zone if symptoms should reoccur.

**Figure 3:**
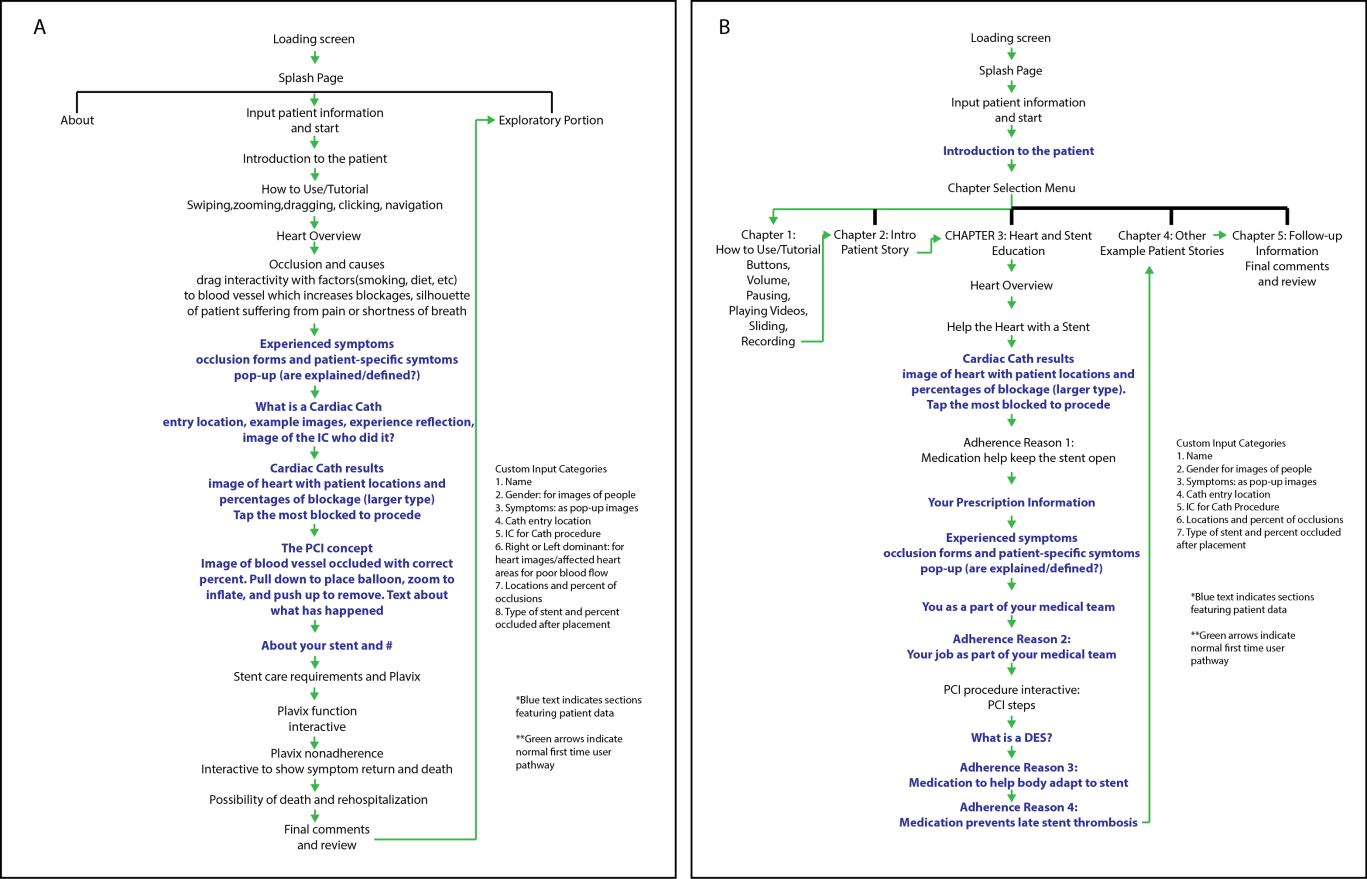
Outlines of the My Interventional Drug-Eluting Stent Educational App learning module. (A) Initial outline. (B) Revised outline based on feedback and closer adherence to Kolb’s theory.

**Figure 4:**
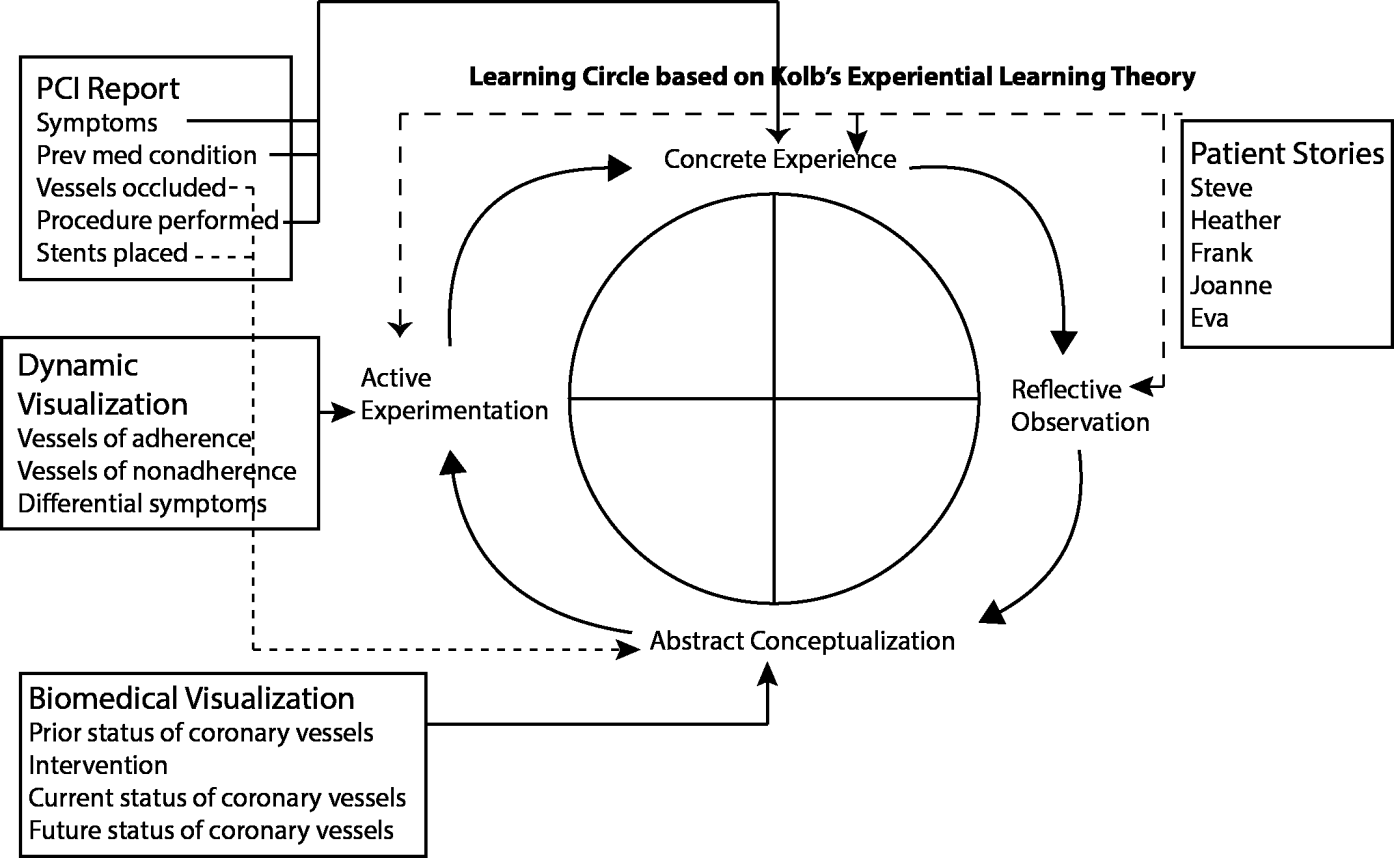
Conceptual framework for the My Interventional Drug-Eluting Stent Educational App. Integration of the multiple aspects of the project with Kolb’s experiential learning theory. The patient will start at the concrete experience of the symptoms of the disease and patient stories. The patient will then reflect on the patient stories. The biomedical visualization pulls in information from the percutaneous coronary intervention report and provides the learning content (abstract conceptualization). Finally, the active experimentation allows the patient to see the potential outcomes of failure to adhere to the suggested medication regimen.

The updated app outline had 5 chapters and text rewritten to reflect the purposes of the DAPT (Figure 3). The specific program features were mapped to the Kolb’s experiential learning theory (Figure 4). Because of scheduling challenges, the patient advisers and the multidisciplinary team separately discussed the subsequent third wireframe. The patient stories developed from the prior comments focused on the following 5 reasons for medication discontinuation: (1) exhaustion after hospitalization, (2) information about duration of medication, (3) cost of medication, (4) travel and challenges of refills, and (5) side effects (Figure 4). Only 2 issues emerged from the discussions concerning the third wireframe: the need for an additional feature, a replay audio button in case someone wanted to hear the audio again, and the need to increase the size of the buttons and controls. Patient advisers focused on giving input about certain slides saying, “Since there are more recorded/narrative sections later on, is there a need to insert an audio practice example at this point (slide 23)? This will help validate clarity of patient’s speech and possibly make him/her adjust his/her voice for maximum playback quality.”

Although additional meetings occurred for iterations 4-6 of the wireframes, most of the suggestions were about the educational level of the language, layout, and text phrasing; no additional major functional issues emerged. MyIDEA was designed for patients with a sixth-grade reading level so that most patients could understand the information. The patient advisers suggested sentences be shorter, which are conducive to a sixth-grade reading level. Advisers also concluded that if the patients have less than a sixth-grade reading level, the audio track and images will help supplement the written text.

When a hard copy of the final wireframe was shown to 3 IRB-approved consented research participants in a hospital setting, their feedback was extremely positive. They were excited and appreciative to learn more about their PCI procedure. They also liked the idea of patient stories and being able to relate and learn from others in understandable language, through the audio component and graphics of those going through the same experience as themselves.

**Figure 5:**
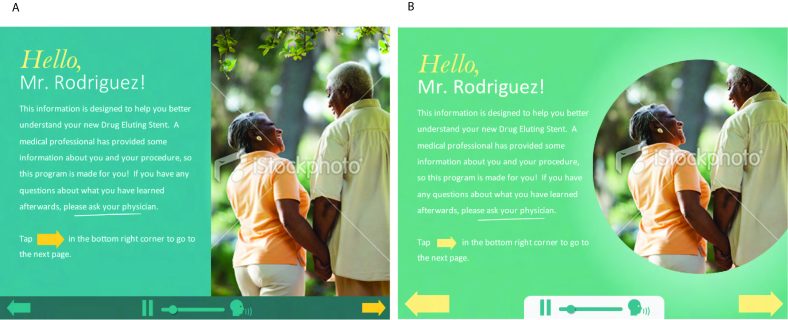
Color design concepts of the My Interventional Drug-Eluting Stent Educational App: (A) Concept 3 and (B) Concept 4.

The seventh wireframe focused on information simplicity and clarity for the patient. The buttons were located at the bottom of the screen, so the patient would focus on the content, not the buttons. One patient adviser said, “Using the program is essential; do not skip the introduction. The patient needs to know what all the buttons are for and where to call or email for program assistance. Make this as simplistic as possible because many patients many not be computer literate. Most may dislike computers and have been fighting it for years. The key is getting the patients to follow the postcardiac instructions.” Of the 2 color designs, the patient advisers were concerned with the text being difficult to read. Another concern was the font size. MyIDEA color design concept 2 was simpler but was described as “not eye-catching.” Another patient adviser stated, “Make this pleasing and eye-catching. It needs to have a positive vibe, so shades of yellow, green, or roman red can be used to create a continuum.” MyIDEA color design concept 2 also incorporated the color palettes of a sports team the patient advisers favored. Patient advisers strongly recommended colors that were bright, as well as common color combinations. MyIDEA color design concept 3 and 4 were created (Figure 5) as a result and patient advisers suggested using MyIDEA color design concept 3. After a few final touches improved the concept for the final design, the patient advisers said it was innovative, and easy to follow.

### Data Storage

After final design approval of MyIDEA, the programming of the databases, app servers, and the Android app began. The University of Illinois has a policy that prohibits the storage of personal health information on electronic mobile devices. As a result, the app has been designed to load a patient’s individual information only when loading the app through an encrypted communication to the server where the data are stored. The patient’s data are inserted into the app upon entry; however, when the app is exited, all data are purged from the tablet. All audio recording and interactivity with the program are recorded and sent through an encrypted connection back to the server.

### Final Design and Deployment

After the MyIDEA program was complete, the team evaluated the fully interactive and functional app. A common challenge arose in the ability to click with the tablet. The challenge with the app was that the test users were unable to advance to the next screen. The inability to advance to the next screen was the limited definition of a click on the tablet. What many individuals considered a click was read by the tablet as another advanced feature on the tablet. The Android app was thus reprogrammed, enabling any touch-screen action by the advance arrow button to be interpreted as a single click. In addition, it was also noted that because elderly patients often better understand lower-pitched, male voices, it would be beneficial to include such a voice option as well. Additional feedback was directed at the narrations and certain portions were rerecorded to ensure proper pronunciation. Other text was reworked to account for the presence or absence of bare metal stents when multiple stents are used.

To facilitate the patient’s reflection (Figure 4), the MyIDEA app asks questions and records the answers through the tablet’s microphone. The recording starts with a simple click of a recording icon. Playback of the recording is an option available to the patient.

The logistics of when and where the patient-centered education would occur was discussed with the patient advisers. It was noted that the patient would not be very attentive shortly after their procedure because of stress related to the hospitalization or medications. It was determined that for the MyIDEA app to enhance medication adherence, it would likely be more advantageous to have this education a few days after the procedure. Based on this feedback, the MyIDEA app is offered both in the hospital setting for the first visit, which is right after the procedure, and again at the follow-up appointment in 1-3 weeks, which is scheduled for the same day as the research participants’ follow-up appointment with their cardiologist. Clinical research nurses approached the patients for consent. Upon consent, the clinical research nurses present the MyIDEA program to the research participant randomized to the intervention. The clinical research nurses are also present for the second visit. This app is used as an educational tool in which the participants interact with the tablet app to learn more about their procedure and postprocedure medication plan. All research questions are directed to the clinical research nurses who are on-site. Participants are advised to contact their physician or clinical nurses should they have any questions about their stent or postprocedure drug adherence issues. The app has slides that reiterate this point.

The MyIDEA program is deployed in a pilot randomized control trial that is ongoing. This randomized study had an inclusion criterion of a patient who received at least one DES. Recruitment occurred after the PCI procedure, and after the cardiologist had placed the type of stent he/she decided would be used. The study has a control arm, which consists of normal physician and nurse education that all patients receive, and an interventional arm exposing the participants to theMyIDEA program in the hospital and at the follow-up appointment. A total of 14 participants have consented to be in the trial; 6 participants have been randomized to the educational arm and successfully completed the app in the hospital and again during their second visit. The initial acceptance of this program is a critical aspect of the design methodology. The average time it takes to complete the MyIDEA tablet app, from beginning to the end, is 17.6 ± 3.2 minutes (Table 1).

**Table 1:** Average time taken to complete the MyIDEA app.

	Initial educational intervention (minutes)	Second educational intervention (minutes)
Participant 1	18.8	14.5
Participant 2	16.3	15.7
Participant 3	14.3	14.7
Participant 4	24.9	N/A
Participant 5	20.2	18.6
Participant 6	18.1	N/A

## Discussion

### Development of the MyIDEA App

The involvement of the patient advisers in the development of the MyIDEA app was integral to its success. While it is tempting to wait to seek users or patient advice until a finished product is available, the insight and critique early in product development helped in ways that could not have been envisioned at the beginning of the process. Despite their lack of software development experience, the patient advisers provided valuable comments and insight, which were vital to the development of MyIDEA. Because the patient advisers had been cardiac patients themselves, it was a beneficial experience to have them see the new technology as it was developed from a patient’s point of view. Some of the ideas that patient advisers focused on were color choice, word usage, and ease of use of the app. Team members readily added their own analysis on color scheme, creating a reflective interaction between collaborators. In the layout and design phase of MyIDEA, patient population attributes were integrated in the early stages [30]. There were 7 wireframes that were created before the final tablet app was finalized. Elements such as lines, boxes, colors, and text were altered after consultation with both the health professional team and the patient advisers.

Kolb’s experiential learning theory was used to create the framework for the multidimensional educational tablet app using the following 4 stages: concrete experience, reflective observations, abstract conceptualization, and active experimentation [13]. There was use of concrete experience with the slides focused on patient stories and having the research participants respond to questions about symptoms of their disease. Reflective observation was used as the participants chose which of the patient stories they most related to and then solved the newly presented concerns raised in those stories. Abstract conceptualization was integrated into slides with a visual depiction about vessel blockage before and after the operation for the research participants to learn about the importance of DES and medication adherence using their PCI report to tailor the program to each individual. Active experimentation was used in the app for research participants when slides focused on how other patients overcame the obstacles in the stories and to apply that information to themselves.

In addition, this educational, interventional tablet app can be classified in terms of behavioral change techniques (BCTs) [31]. A structured taxonomy has been established to categorize BCTs used in behavior change interventions [31]. This particular tablet app includes features from 7 of the 16 known clusters. A total of 8 BCTs were analyzed within the 7 clusters that showed how this tablet app is used to change behavior such as increase medication adherence. Use of behavior rehearsal/practice, covert conditioning, problem solving/coping planning, review of outcome goals, modeling of the behavior, mental rehearsal or successful performance, comparative imagining of future outcomes, and instruction on how to perform a behavior were the 8 BCTs that this tablet app targeted. These techniques are shown to modify behavior and, in this study, are expected to increase medication adherence after the surgical procedure. Within the biomedical communication literature, collaborative research is the norm [29]. During discussions, researchers indicated that the engagement of patient evaluators is critical; however, they only obtain patient evaluation of a finished product [32] or for insight into the amount of visual noise in images [29]. In this study, patient advisers were involved from an early stage, which is unique to the experience of the biomedical communication community. The use of multimedia and images with a multimedia approach has resulted in increased learning of similar content compared with traditional text for patient education [33]. The MyIDEA development of dynamic customized content expands on these concepts.

Within the health informatics literature, eHealth interventions have been shown to be promising compared with normal treatment [27]. However, in that review only 12 interventions were evaluated [27]. As with MyIDEA, the vast majority of interventions were derived from a health behavior theory [27]. The health literature also stresses the importance of tailoring care to the patient [34]. The MyIDEA app is customized, reflecting the patient’s procedure findings, symptoms, and prescriptions.

Three main concepts of participatory design have been discussed in computer science literature: the politics of design, nature of participation, and methods [25]. The politics of MyIDEA are unique in that multiple health professions and patients all came together for a common goal. The professional domains and experience were leveraged to have all participants contribute equally to ensure the project was a success. The nature of participation was unique compared with other software development projects. Participation by faculties and patient advisers was voluntary, because no grant money was available at the time of development. The development of MyIDEA was done using internal departmental funds with no salary for either the faculties or patient advisers. After completion of a fully functioning app, the National Institutes of Health pilot grant money enabled us to enroll research participants. The app was completely debugged before the funding was received. If a member of the development team decided not to fully participate, they were able to leave at any point in the development process. The method of participation was regular meetings with prototyping.

Our approach was consistent with the ISO standard 9241-210:2010, which was a revision from the 1999 standard [26]. One of the revisions included in this standard was that human-centered methods could be used throughout the system life cycle [26]. The use of patient advisers from the initial wireframe of MyIDEA is following the reimaged technical specifications of the ISO standard. A detailed analysis of the over 30 different parts of the ISO standard are beyond the scope of this paper. We avoided conventional methodology such as Patient Education Materials Assessment Tool and Assessing the Quality of Decision Support Technologies Using the International Patient Decision Aid Standards instrument because this study focuses on the unique challenge of postprocedure education [35,36]. Other evaluation tools focus on checklists. No checklists were used before the PCI procedure as it is unknown whether a patient will have a DES or a BMS.

### Conclusions

The patient-centered educational program, MyIDEA, incorporates patient-specific information for tailoring. Patient adviser participation in the early phases of mobile patient education development is a critical key to the success of the intervention. From the initial response of the patients, the MyIDEA app is ready for efficacy trials. The future of patient education will have the expectation to include tailored information to enhance the overall quality of care. Multidisciplinary literature, collaborative design, and patient engagement early in the design are critical for success. Patients are sophisticated consumers, who desire knowledge about their health and procedures to help make informed choices. Thus, working with patient advisers to help design and present information for consumption is an invaluable process. Each patient adviser had a postcardiac experience and was well aware of the questions, anxiety, and need for understandable information in a form that would be rapidly available. Mobile patient education has the potential to transform health care; however, without *early* patient participation the potential of this new technology will remain unfilled.

## Abbreviations

BCT: behavioral change technique
DAPT: dual antiplatelet therapy
DES: drug-eluting stent
IRB: Institutional Review Board
ISO: International Standardization Organization
MyIDEA: My Interventional Drug-Eluting Stent Educational App
PCI: percutaneous coronary intervention
